# Editorial: The interactional role of host and microbiota in diseases and intervention strategies of traditional Chinese medicine

**DOI:** 10.3389/fcimb.2023.1253970

**Published:** 2023-08-21

**Authors:** Tianhao Liu, Lianlin Su, Ya Xiao, Yilin Ren

**Affiliations:** ^1^ Department of Gastroenterology, Affiliated Hospital of Jiangnan University, Wuxi, China; ^2^ College of Pharmacy, Nanjing University of Chinese Medicine, Nanjing, China; ^3^ College of Chinese Medicine, Jinan University, Guangzhou, China

**Keywords:** interactional role, host, microbiota, intervention strategies, traditional Chinese medicine

The interaction mechanisms between gut microbiota (GM) and the host are becoming more obvious as science and technology improve. The composition and structure of the GM have all been related to the occurrence, development, and prevention of various diseases, including tumors, inflammatory bowel disease, diabetes, and others. Traditional Chinese medicine (TCM) has made significant advances in gut microbiome research, showing a strong link between GM and human health and providing many treatment alternatives. The following discussion will center on the topics of the 12 most recent important research in our area, which will investigate the potential and prospects of TCM in the field of the GM.

*Soybean isoflavones* have been found to modulate the GM, resulting in health benefits for weight management and metabolism. Researchers have observed that the consumption of *soybean isoflavones* can positively influence the composition of the GM, leading to improved metabolic functions and weight control (Huang et al.). Baohe pill decoction has showed potential in treating diarrhea caused by a high-fat, high-protein diet. It has been found that the efficacy of Baohe pill decoction is associated with the structure of the lactase-producing bacterial community in the gut. By targeting and modulating bacterial community, Baohe pill decoction helps restore normal digestion and reduce diarrhea symptoms (Zhou et al.). Researchers have conducted a comprehensive evaluation of the mechanism by which *Gastrodia elata Blume* ameliorates cerebral ischemia-reperfusion injury. Through the integration of fecal metabolomics and 16S rDNA sequencing, they discovered that *Gastrodia elata Blume* acts on the GM, leading to neuroprotective effects. This research provides valuable insights into the therapeutic potential of *Gastrodia elata Blume* in treating cerebral ischemia-reperfusion injury (Ding et al.). There has been significant progress in studying the GM of ethnic minorities in China. Researchers have explored the differences in gut microbial composition among different ethnic groups and identified potential intervention strategies based on traditional ethnic medicine. Understanding the role of GM in ethnic minorities can help develop targeted interventions to improve their overall health (Chen et al.). One of the researches shows that the gut microbiome can influence the metabolism of Taohong Siwu Decoction (TSD), a traditional Chinese herbal formula used in glioma treatment. By elucidating the interactions between the GM and TSD, researchers can improve their understanding of the formula’s therapeutic effects on glioma (Feng et al.). Not come singly but in pairs, Baihu Renshen Decoction, a traditional Chinese herbal formula, has been found to ameliorate type 2 diabetes mellitus in rats. This beneficial effect is attributed to its ability to modulate the GM, enhance gut permeability, and inhibit TLR4/NF-κB-mediated inflammatory responses (Yao et al.). These findings highlight the potential of using TCM to target the GM for the treatment of diabetes. The gut-brain interaction theory has shed light on the connection between the GM and neurogenesis. TCM research can leverage this understanding to explore the role of GM in neurological disorders and develop novel therapeutic strategies (Zhang et al.). Dendrobium officinale, a commonly used herb in TCM, has been shown to alleviate nonalcoholic steatohepatitis induced by a high-fat diet. This effect is linked to its ability to modulate the GM. By targeting the GM, *Dendrobium officinale* offers a potential approach for the prevention and treatment of liver diseases (Tian et al.). As is known to all, improper diet combined with high temperature and humidity environments can lead to alteration of intestinal mucosal microbiota and result in diarrhea with Chinese dampness-heat syndrome. Understanding how these factors impact the GM can provide insights into the underlying mechanisms and guide the development of effective interventions (Qiao et al.). *Bletilla striata polysaccharides and oligosaccharides* have been found to undergo digestion and fermentation by human GM. This research emphasizes the role of GM in metabolizing plant-based compounds and highlights the potential health benefits associated with the consumption of *Bletilla striata* (Wang, Q. et al.). Drug resistance has always been a thorny issue that troubles doctors and patients, and TCM has a good intervention effect in this regard. TCM therapy based on the manipulation of the GM offers a new approach to overcoming antibiotic-resistant bacteria. By modulating the GM, TCM can enhance the effectiveness of antibiotics and reduce the emergence of resistance (Xue et al.). Non-small cell lung cancer (NSCLC) can be approached from a GM perspective in TCM. The GM has been found to play a significant role in NSCLC development and progression. Understanding this link can potentially lead to the development of personalized treatments for NSCLC patients (Wang, X. et al.).

Existing evidence suggests that the GM appears to play an important role in the occurrence and development of tumors, inflammatory, and metabolic diseases. However, more research is needed on the physical, chemical, and biological roles of GM in drug intervention or treatment of the aforementioned diseases. Our research theme mainly reveals that Chinese herbal medicine or its single compound can significantly regulate GM, and preliminarily reveals the interaction between GM and host, providing strong evidence for the intervention strategy of TCM. In summary, the studies discussed in this Research Topic highlight the important role of the GM in various health conditions and the potential for TCM to modulate and utilize the GM for therapeutic purposes. In the future, the unique role of GM, such as “the messenger of Chinese medicine ingredients”, “processing factory”, “GM energy supply station” and other special roles, would require new and future topics to be exposed and explored. The future dual-use food of TCM and food will be a whole new business in terms of industrial change. Due to the complex chemical components contained in traditional Chinese prescriptions, it is difficult to directly or completely reveal the material basis or mechanism of action of traditional Chinese prescriptions during the research process. Therefore, researchers need to conduct in-depth research on it from different perspectives. Thus, the study of the effective ingredients of TCM based on sterile animals is a new method to reveal the relationship between GM and medical compounds. Finally, understanding the interaction between the GM and TCM offers up new options for study and the development of novel therapies. Further research into this topic has considerable promise for enhancing human health and well-being.

## Author contributions

TL: Conceptualization, Funding acquisition, Investigation, Project administration, Writing – original draft, Writing – review & editing. LS: Investigation, Methodology, Writing – original draft, Writing – review & editing. YX: Software, Writing – review & editing. YR: Writing – original draft.

